# SARS-CoV-2 and the Gastrointestinal Tract in Children

**DOI:** 10.3389/fped.2021.617980

**Published:** 2021-02-22

**Authors:** Maria Giovanna Puoti, Anna Rybak, Fevronia Kiparissi, Edward Gaynor, Osvaldo Borrelli

**Affiliations:** Department of Pediatric Gastroenterology, Great Ormond Street Hospital, London, United Kingdom

**Keywords:** SARS-CoV-2, COVID-19, gut, gastrointestinal symptoms, children, pediatric gastroenterology

## Abstract

Coronavirus disease 2019 (COVID-19), caused by acute respiratory syndrome coronavirus 2 (SARS-CoV-2), is predominantly a respiratory disease. However, its significant impact on the gastrointestinal (GI) system is now well-known. SARS-CoV-2 enters cells *via* the angiotensin-converting enzyme-2 (ACE-2) receptor, which is abundantly expressed on lung cells, but also on enterocytes. Several etiopathogenetic mechanisms have been postulated to explain the GI involvement in COVID-19, including loss in intestinal absorption, microscopic mucosal inflammation and impaired ACE-2 function, which plays a significant role in maintaining gut homeostasis. In children the GI manifestations include anorexia, nausea, vomiting, diarrhea and abdominal pain, which may represent the earliest presenting symptoms of the disease. However, although rare, a significant GI mucosal inflammation, such as terminal ileitis mimicking an atypical appendicitis, and other GI manifestations have been reported. COVID-19 pandemic has posed a significant challenge in healthcare provision in term of ability in providing safe diagnostic procedures, face-to-face consultations, and offering comprehensive care. For instance, changes in health services have raised the risk of empirical or sub-optimal management of chronic GI disorders such as inflammatory bowel disease (IBD) due to delayed endoscopic and clinical assessment. This review will discuss the acute GI involvement in COVID-19 in children and reflect on challenges and major changes observed in clinical practice during COVID-19 pandemic by sharing both the published literature and personal experience. We also suggest potential strategies for providing optimal gastroenterology care during this unprecedented era.

## Introduction

The severe acute respiratory syndrome-coronavirus-2 (SARS-CoV-2) was first identified in patients with a severe form of pneumonia in Wuham province in December 2019 (1). Since its global spread, SARS-CoV-2 has been recognized as the etiological agent of coronavirus disease 2019 (COVID-19), the presentation of which ranges from asymptomatic or mild respiratory symptoms to severe lung injury, multi-organ failure and death. Although it is commonly known as a respiratory illness, it is now evident that it can also affect the gastrointestinal (GI) system, with highest incidence in pediatric age.

We review gastrointestinal disorders in children with COVID-19 and hypothetical mechanisms leading to gut symptoms, and discuss the clinical implication for pediatric gastroenterology services and impact on chronic gastrointestinal diseases.

## Materials and Methods

A systematic search and review of literature was conducted following the PRISMA guidelines using online databases PubMed, Embase, Scopus, Medline and Google Scholar for the period between January 1st 2020 and November 23rd 2020.

The key words for the search were SARS-CoV-2, COVID-19, gut, bowel, intestine, digestive, gastrointestinal, gastroenterology, child, children, infant, adolescent, pediatric, and pediatric. The following search term were used in Pubmed to set the first dataset of articles: ((“severe acute respiratory syndrome coronavirus 2”[Supplementary Concept] OR “severe acute respiratory syndrome coronavirus 2”[All Fields] OR “ncov”[All Fields] OR “2019-nCoV”[All Fields] OR “COVID-19”[All Fields] OR “SARS-CoV-2”[All Fields] OR ((coronavirus[All Fields] OR “cov”[All Fields]) AND 2019/11[PubDate]: 3000[PubDate])) AND (“gut”[All Fields] OR (“intestines”[MeSH Terms] OR “intestines”[All Fields] OR “bowel”[All Fields]) OR (“intestines”[MeSH Terms] OR “intestines”[All Fields] OR “intestine”[All Fields]) OR (“digestive system”[MeSH Terms] OR (“digestive”[All Fields] AND “system”[All Fields]) OR “digestive system”[All Fields] OR “digestive”[All Fields] OR “digestion”[MeSH Terms] OR “digestion”[All Fields]) OR gastrointestinal[All Fields] OR (“gastroenterology”[MeSH Terms] OR “gastroenterology”[All Fields]))) AND ((“child”[MeSH Terms] OR “child”[All Fields]) OR (“child”[MeSH Terms] OR “child”[All Fields] OR “children”[All Fields]) OR (“pediatrics”[MeSH Terms] OR “pediatrics”[All Fields] OR “pediatric”[All Fields]) OR (“pediatrics”[MeSH Terms] OR “pediatrics”[All Fields] OR “pediatric”[All Fields]) OR (“infant”[MeSH Terms] OR “infant”[All Fields]) OR (“adolescent”[MeSH Terms] OR “adolescent”[All Fields])) AND (“2020/1/1”[PDat]: “2020/11/23”[PDat]).

Duplicates were removed and a first filtering was applied based on the following exclusion criteria: non-english manuscripts, no-full text available and no pediatric cases included in the studies. The eligibility of remaining pool of articles was screened based on their title and relevance of abstract to the main topic of the present review. Studies meeting all the following criteria were included: (a) reported gastrointestinal symptoms in COVID-19 pediatric population; (b) reported impact of COVID-19 pandemic on pediatric chronic gastrointestinal disorders; (c) reported impact of COVID-19 pandemic on pediatric gastroenterology practice; (d) full-text article that were peer-reviewed; (e) published in english.

In order to guarantee the comprehensiveness and accuracy of our research, we also reviewed the references of included articles by using the same screening criteria as the first search, so that the snowball method was used to identify additional papers that were missed by the systematic literature search.

**Figure 1:**
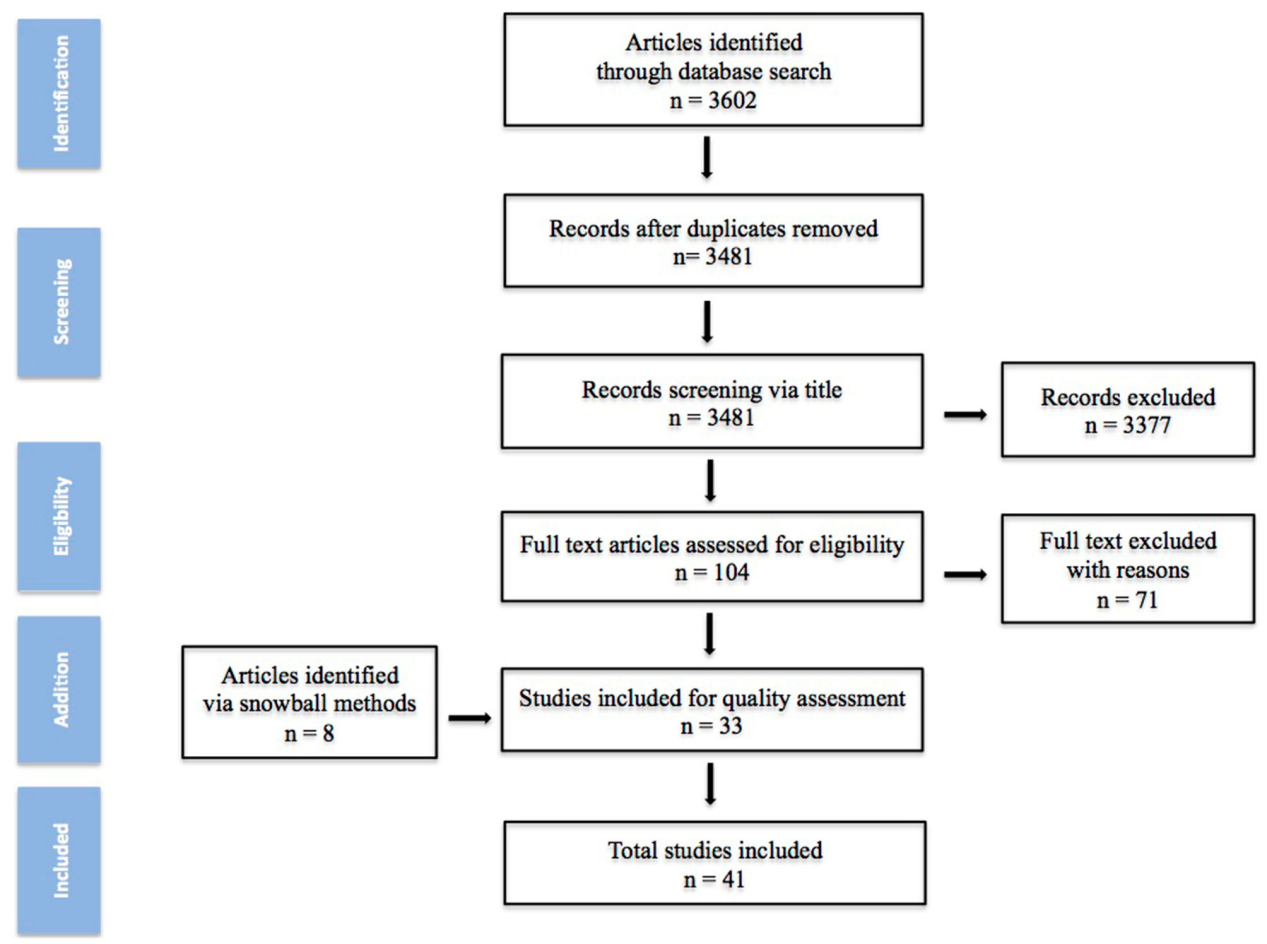
Methodology for literature review.

Based on the previous search strategy, a total of 3,602 studies were obtained from the database. After deletion and screening process, 104 articles were elected for full-text assessment and 8 added *via* snowball process. Finally 41 studies were included in this review. The quality assessment of the studies is summarized in Figure 1.

## SARS-CoV-2 and Gastrointestinal Tract

### Gastrointestinal Tract as Target of SARS-CoV-2

SARS-CoV-2 is a novel RNA β-coronavirus and its full-genome sequencing has defined its belonging to the same subgenus of the Severe Acute Respiratory Syndrome (SARS) virus and several bat coronaviruses. The original source of the virus is unclear, although it is likely to originate from an animal reservoir, as reported for other coronaviruses (2).

Similar to influenza, SARS-CoV-2 RNA transmission largely occurs through droplets spread, and from hand to mucous membranes, such as eyes, nose and mouth, although it has been also detected in non-respiratory specimens, such as stool, blood, ocular secretions, semen, and breast milk (3). Hence other routes of transmission have also been postulated, although their role in the viral transmission remains unknown (4).

SARS-CoV-2 enters host cells *via* the binding of the viral spike (S) glycoproteins to the angiotensin-converting enzyme-2 (ACE-2) receptor, which is highly expressed on type I and II alveolar epithelial cells in the lungs (5). Involvement of ACE-2 receptor in lung parenchyma is thought to be important both in disease severity, and subsequent onwards transmission (6). ACE-2 is a homolog of angiotensin converting enzyme, the central enzyme in the renin-angiotensin-aldosterone system, which plays a crucial role in the physiology and pathology of all organs (7). After binding ACE-2 receptor, virus entry requires cleavage of the spike protein (priming) by a type II transmembrane serine protease (TMPRSS2). This priming step is essential for fusion of the viral and cell membranes (5, 8, 9). It has been suggested that additional proteases, including furin, might also be involved in SARS-CoV-2 priming (10). Hence, the expression of ACE-2 is essential for SARS-CoV-2's entry into host cells. In humans, ACE-2 and TMPRSS2 are strongly expressed in lung tissue and in particular by epithelial cells, which explains why the lungs appear to be the most vulnerable target during the infection. ACE-2 is also expressed in many extrapulmonary tissues, including GI tract, heart, liver, and kidney (11–13).

High levels of ACE-2 receptors have been found on the luminal surface of differentiated epithelial cells in the small intestine, whilst levels are lower in the crypt cells and colon (13, 14). It is likely that SARS-CoV-2 replicates within the GI tract through the ACE-2 receptor on intestinal enterocytes. ACE-2 is known to have several roles within digestive system, including absorption of amino acids and maintenance of gut homeostasis (13, 15). It has been postulated that the GI tract might be a major entry site for SARS-CoV-2, raising the possibility that the virus could propagate in humans *via* fecal-oral route. SARS-CoV-2 has been detected in fecal samples of COVID-19 patients, suggesting that the GI tract might be a site of viral replication and activity (9, 16). Around half of all COVID-19 patients have detectable viral RNA in their stools, even when it is no longer identified in the respiratory tract (16–18). Viral shedding of SARS-CoV-2 has been demonstrated in stool for almost 5 weeks beyond the first negative nasopharyngeal aspirates in children diagnosed with COVID-19, as part of a prospective screening cohort of COVID-19 contacts (19). SARS-CoV-2 RNA has also been found in the feces of the majority of COVID-19 patients and asymptomatic carriers up to 30 days after symptom onset, even after negative respiratory swabs (19, 20). Moreover, the virus has also been detected in GI histological samples obtained during endoscopy (21, 22). In a single center series of 95 patients, of whom 61% had GI symptoms and 6 underwent endoscopy, SARS-CoV-2 was detected in the esophagus, stomach, duodenum and rectum of the two most unwell patients, and in the duodenum in one of the other patients (22). However, pathogenicity of fecal SARS-CoV-2 RNA detected in fecal samples should be interpreted with caution as by using reverse-transcriptase polymerase chain reaction (RT-PCR) techniques, viable viral isolation and culture have not been shown in stool (23).

Nonetheless, as other human coronavirus, SARS-CoV have been shown to be infectious in laboratory conditions up to 2 weeks after samples were taken from sewage from hospital treating patients with SARS (24). Several studies using human small intestinal organoids have shown that SARS-CoV-2 replicates in enterocytes (9, 25, 26). In a mouse model, intragastric inoculation of SARS-CoV-2 was able to provoke productive infection and lead to pulmonary pathological changes, suggesting a potential pathological impact of the fecal-oral route (27). To date, whether the intestinal epithelial cells are primarily infected with SARS-CoV-2 *via* the oral–fecal route or the enteric infection is secondary to respiratory infection is still unclear. Further studies assessing the risk of COVID-19 fecal-oral route transmission are clearly warranted.

### Pathogenesis of Gastrointestinal Disorders in SARS-CoV-2 Infection

Several etiological factors seem to be involved in the pathogenesis of GI involvement in COVID-19 patients.

A potential cross-talk between lung and gut defined as “lung-gut axis,” has been postulated in SARS-CoV-2 infection. The cytokine storm caused by a hyperactive host immune system increases release of inflammatory mediators lead to lung hyper-permeability such as the virus along with the inflammatory mediators *via* circulation migrates to intestine and binds ACE-2 receptors on the enterocytes (28).

The activation of innate and adaptative immunity with recruitment of inflammatory cells and local and systemic production of inflammatory cytokines in the intestinal epithelium are likely to play a role. The expression levels of genes encoding human type III interferons (INFs), IFNλ2, and IFNλ3, are significantly induced in the infected enteroids. Similarly, a significant upregulation of the genes encoding C-C chemokine receptors (CCR1 and CCR8), genes encoding interleukines (IL) 16 (IL16), IL3, and C-X-C motif chemokine ligand 10 (CXCL10), also known as interferon gamma-induced protein 10 (IP10), is also observed. Conversely, genes encoding CCR2, CCR5, and IL5 are downregulated, whilst, upon infection, those encoding IFN-α, IFN-β, and IFN-γ are barely induced. The effect of regulation of these antiviral and inflammatory mediators on viral replication and host response requires further clarification (9). Possibly the immune response could lead to a dysbiosis with a propagation of the pro-inflammatory state. Bacterial translocation may be an early event related to intestinal damage due to tissue infection, systemic inflammation-induced dysfunction and IL6-mediated vascular damage. It has been suggested that SARS-CoV-2 infection alters the gut-blood barrier, leading to systemic dissemination of bacteria, endotoxins, and microbial metabolites (18, 29, 30). Moreover, local inflammation might weaken the mucosal barrier as well as viral replication and spreading might contribute to futher epithelial damage and mucosal inflammation, which in turn could be responsible of the development of GI symptoms in COVID-19 patients. It has been shown that the virus infects intestinal epithelial cells and instigates an acute mucosal inflammation response as confirmed by an elevated fecal calprotectin (31).

GI symptoms in COVID-19 patients might also result from dysfunction of ACE-2, a key regulatory enzyme in the renin-angiotensin-aldosterone system, which is known to modulate gut immune functions and inflammation (32). Disruption of ACE-2 function affects the intestinal homeostasis making the GI tract more susceptible to inflammatory process. For instance, it has been shown a decreased uptake of ingested tryptophan in ACE-2 knockout mice, which leads to a decrease in the expression of antimicrobial peptides in small intestinal paneth cells and a change of the intestinal microbiota, and in turn to the development of colitis (13). By analyzing fecal sample using 16S rRNA gene sequencing, in a cohort of 30 COVID-19 patients the infection was associated with a decrease in bacterial diversity and abundance, with a reduction in beneficial bacteria (33).

The susceptibility to the SARS-CoV-2 infection may be influenced by the presence of ongoing mucosal inflammation and immune dysregulation, which characterized some chronic GI disorders. For instance, ACE-2 and TMPRSS2 receptors were found to be upregulated in inflamed intestine of inflammatory bowel disease (IBD) patients, which might hint at an augmented susceptibility to SARS-CoV-2 (34, 35). On the other hand, it has been suggested that the increased serum concentration of soluble form of ACE-2, documented in IBD patients, might exert a protective role against the infection by acting as a competitive receptor for the virus (34).

Recent evidence suggests that SARS-CoV-2 may also affect the central nervous system (CNS), which exerts a key role in the gut-brain axis (36, 37). ACE-2 and TMPRSS2 have also been detected in the human enteric nervous system (ENS) of both small and large intestine suggesting the possibility of a neurogenic transmission. It has been postulated that the infected ENS could serve as direct entry for CNS viral neuroinvasion *via* the vagus or splanchnic nerves (38). However, it is unknown whether this might modulate the function of the enteric nervous system, leading to symptoms such as pain or altered bowel habit. Moreover, the role of SARS-CoV-2 on the disorders of gut-brain axis, such as functional GI disorders (FGIDs), and the role of ENS infection in developing GI manifestations during COVID-19, is matter of debate.

**Figure 2:**
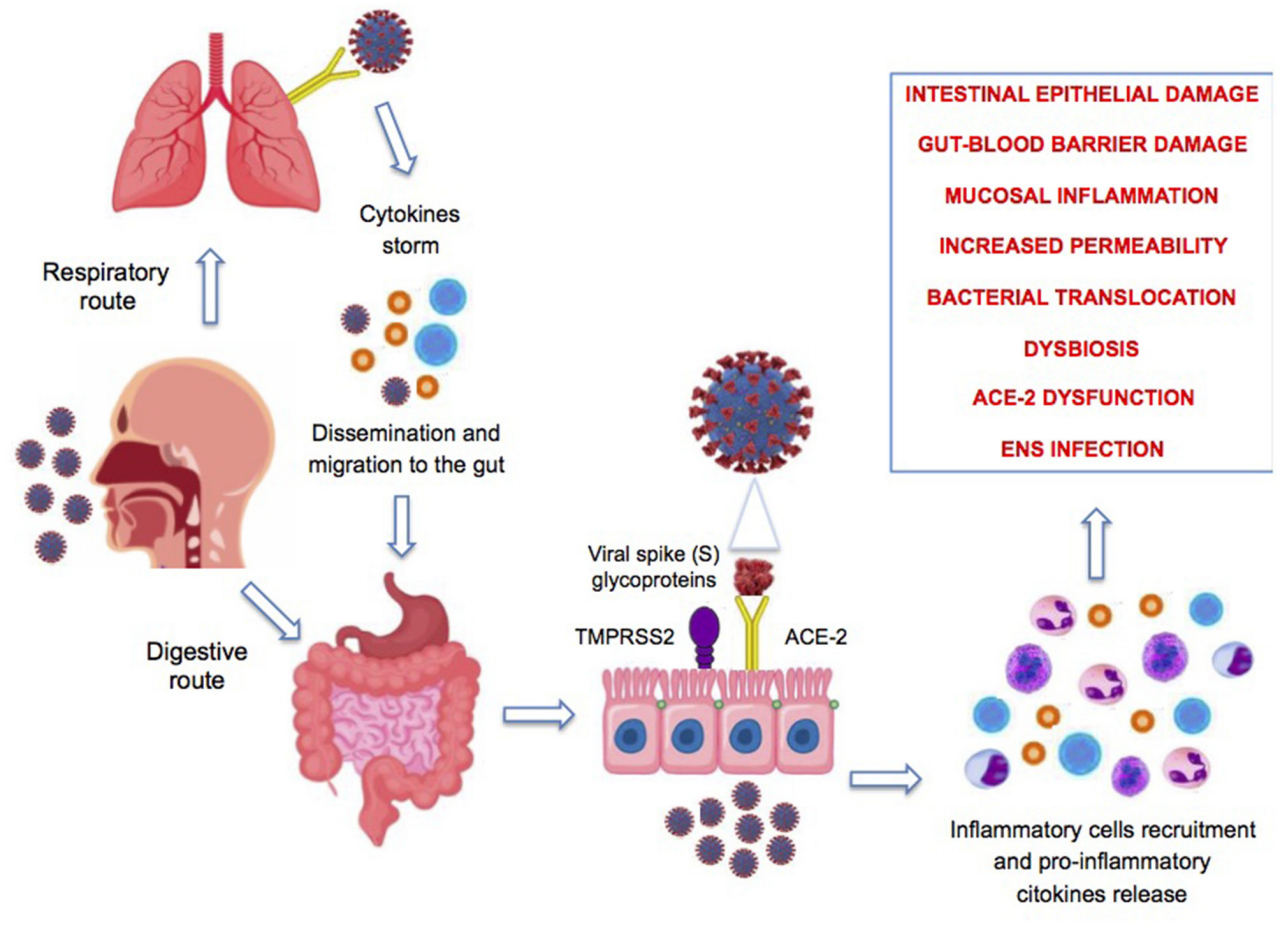
Proposed pathophysiologic mechanisms of gastrointestinal symptoms associated with COVID-19 infection. ENS, enteric nervous system; ACE-2, angiotensin-converting enzyme-2.

Figure 2 summarizes the potential mechanisms involved in the pathogenesis of intestinal damage during SARS-CoV-2 infection.

### Gastrointestinal Symptoms of SARS-CoV-2 Infection in Children

COVID-19 is documented to have a wide spectrum of symptoms, with clinical difference at presentation between adults and children. In adults, pneumonia appears to be the most frequent and serious manifestation of infection, characterized primarily by fever (94%), cough (79%), dyspnea (31%), and bilateral infiltrates on chest imaging. Symptoms such as fatigue, sore throat, loss of smell and taste and myalgia, have also been reported. Gastrointestinal symptoms such as diarrhea are much less common (5–15%) (39, 40).

It should be noted that the various research groups, which have analyzed the epidemiological and clinical characteristics of COVID-19, have shown a large discrepancy in the prevalence of specific GI symptoms. In several studies performed in China and Hong Kong, the proportion of COVID-19 adult patients developing gastrointestinal disorders ranged between 8.7 and 26% (18, 22, 30, 39, 41, 42). A European study reported a prevalence of 55%, whilst studies from the United State showed a range between 35 and 61% (31, 43, 44). Meta-analyses of studies pooling 4,243, 6,686, and 10,890 patients, respectively, found that the combined prevalence of gastrointestinal symptoms was 17.6, 15, and 10%, respectively (40, 41, 45).

Jin et al. (46) showed in a group of 651 patients prospectively enrolled prior to treatment, that GI symptoms (11.4% of COVID-19 patients had at least one GI tract symptom) lasted for a median of 4 days and appeared to precede the respiratory symptoms. Moreover, the authors showed that the GI symptoms were more frequent in severe/critical cases of COVID-19 (23%) than in mild COVID-19 (8%) (46).

In pediatric population, the most commons reported GI symptoms are nausea, vomiting, diarrhea, abdominal pain, and feeding difficulties during the course of the infection. However, other GI manifestations have been described. Table 1 summarizes all GI symptoms and signs associated with COVID-19.

**Table 1:** Gastrointestinal manifestations reported in children with COVID-19.

Diarrhea
Nausea and vomiting
Loss of appetite and anorexia
Abdominal pain
Acute appenticitis
Plegmonous ileocolitis
Intussusception
Pneumatosis intestinalis
Protein losing enteropathy
Mesenteric adenopathy

A metanalysis including 1,810 pediatric patients all with PCR tested positive for COVID-19 demonstrates a prevalence of GI symptoms of 6% with higher prevalence of fever (55%), cough (45%), and dyspnea (19%) (47).

Consistent with these findings, Ding et al. (48) found a prevalence of GI symptoms such as vomiting, diarrhea or abdominal pain of 7.4% in a pool of 371 children included in 14 studies. It has been observed that GI symptoms, such as diarrhea (9%), vomiting (10%), and feeding difficulties such as anorexia (23%), are quite common at disease presentation, with fever (42%) and cough (49%) being less prominent in comparison to adults (48).

Table 2 summarizes GI symptoms and their prevalence in several pediatric studies included in our literature search. The results reveal a total prevalence of GI symptoms up to 84.1%. Diarrhea is the most common symptom (up to 56.8%), followed by vomiting (up to 50%), nausea (up to 34.3%), abdominal pain (up to 27.3%), and feeding difficulties (up to 23%).

**Table 2:** Prevalence of gastrointestinal symptoms in children with COVID-19.

**Author**	**Journal**	**Number of patients**	**Median years (range or SD)**	**N (%) with GI symptoms**	**GI symptoms**	**D**	**V**	**N**	**A**	**F**
						**(N; %)**	**(N; %)**	**(N; %)**	**(N; %)**	**(N; %)**
Bialek et al. (49)	MMWR Morb Mortal Wkly Rep, 2020	291	<18	NR	D/V&N/A	37 (13)	31 (11)^∧^	31 (11)^∧^	17 (5.8)	0
Cai et al. (50)	J Med Virol, 2020	3	7.7 (2.2–11)	0	0	0	0	0	0	0
Cai et al. (51)	Clin Infec Dis, 2020	10	6.2 (0.3–10.9)	0	0	0	0	0	0	0
de Ceano-Vivas et al. (52)	Arch Dis Child, 2020	58	3 (0.3–12.2)	NR	D	9 (15.5)	7 (12.1)	0	0	0
Derespina et al. (53)	J Pediatr, 2020	70	15 (9–19)	NR	D/V/N	18 (25.7)	24 (34.3)^∧^	24 (34.3)^∧^	0	0
Du et al. (54)	Allergy, 2020	182	6 (3–15)	20 (11)	D/V/A	9 (4.9)	7 (3.8)	0	7 (3.8)	0
Garazzino et al. (55)	Euro Surveill, 2020	168	2.3 (0.3–9.6)	NR	D/V	22 (13.1)	9 (5.4)	0	0	0
Giacomet et al. (56)	Pediatr Infect Dis J, 2020	107	1.6 (0.3–7.9)	26 (24.3)	D/V/A	20 (18.7)	6 (5.6)	0	6 (5.6)	0
Kim et al. (57)	MMWR Morb Mortal Wkly Rep, 2020	224	<18	NR	D/V&N/A	27 (12.1)	69 (30.8)	69 (30.8)	42 (18.8)	0
Lu et al. (58)	N Engl J Med, 2020	171	6.7 (0–15)	NR	D/V	15 (8.8)	11 (6.4)	0	0	0
Miller et al. (59)	Gastroenterology, 2020	44	7.3 (SD 4.9)	37 (84.1)	D/V/N/A	25 (56.8)	18 (40)	8 (18.2)	33 (75)	NR
Moreno et al. (60)	World J Pediatr, 2020	11	5.1 (0.1–15)	NR	D/V/A	3 (27.3)	3 (27.3)	0	3 (27.3)	0
Parri et al. (61)	N Engl J Med, 2020	100	3.3 (0–17.5)	NR	D/N&V/F	9 (9)	10 (10)^∧^	10 (10)^∧^		23 (23)
Qiu et al. (62)	Lancet Infect Dis, 2020	36	8.3 (1–16)	2 (6)	D/V	NR	NR	NR	NR	NR
See et al. (63)	Int J Infect Dis, 2020	4	6.4 (1.6–11)	1 (25)	D	1 (25)	0	0	0	0
Shekerdemian et al. (64)	JAMA Pediatr, 2020	48	13 (4.2–16.6)	1 (2)	NR	NR	NR	NR	NR	NR
Su et al. (65)	Emerg Microbes Infect, 2020	9	3.7 (0.9–9)	0	0	0	0	0	0	0
Sun et al. (66)	World J Ped, 2020	8	4.4 (0.2–15)	NR	D/V&N	3 (37.5)	4 (50)	0	0	0
Tan et al. (67)	J Clin Virol, 2020	10	7.5 (1.1–12.1)	2 (20)	V/A	0	1 (10)	1 (10)	0	0
Waterfield et al. (68)	MedRxiv, 2020	68	10.1 (2–16)	13 (19)	D/V/A	–	–	–	–	–
Xiong et al. (69)	Gut, 2020	244	1.2 (0.3–7.8)	34 (13.9)	D/V/N/A/F	15 (6.1)	23 (9.4)	0	4 (1.6)	8 (3.3)
Xu et al. (19)	Nat Med, 2020	10	7.5 (0.2–15.7)	3 (30)	D	3/30	0	0	0	0
Yu and Chen (70)	Front Pediatr, 2020	44	7 (1–18)	NR	D/N&V	6 (13.6)	8 (18.2)^∧^	8 (18.2)^∧^	0	0
Zheng et al. (71)	Curr Med Sci, 2020	25	3 (0.3–14)	NR	D/V/A	3 (12)	2 (8)	0	2 (8)	0
Zheng et al. (72)	Pediatr Pulmonol, 2020	52	9 (4–12)	1 (1.9)	D/A/F	NR	NR	0	0	NR
Zhu et al. (73)	Pediatr Pulmonol, 2020	10	9.2 (1.7–17)	0	0	0	0	0	0	0

*GI, gastrointestinal; D, diarrhea; V, vomiting; N, nausea; A, abdominal pain; F, feeding intolerance; NR, not reported*.

∧ *nausea and vomiting were considered together*.

Diarrhea most often occured between 1 and 8 days after the disease onset, but in some patients it was the first symptom. It lasted for 1 up to 14 days with a median duration time of 3.3 days with diarrhea appearing watery in one-third of patients.

Interestingly, GI manifestations can be the earliest presenting symptoms of COVID-19, may precede respiratory symptoms or may manifest later during the disease course and in up to 10% of children they may be the only presentation of the disease (56, 58, 61).

The aforementioned data suggest the need to include SARS-CoV-2 infection among the differential diagnosis of acute diarrhea and/or vomiting, at least for the duration of the pandemic.

Rare manifestations of SARS-CoV-2 infection have also been reported in children, with significant mucosal inflammation being seen in the acute phase, with terminal ileitis associated with symptoms of fever and abdominal pain mimicking an atypical appendicitis (74).

A typical acute appendicitis in the setting of SARS-CoV-2 infection has been reported in a case series of 4 children (age range 11–17 years) tested positive for COVID-19 detected by PCR of nasopharyngeal swab. All cases presented with history of 1 up to 4 days of abdominal pain, vomiting and/or nausea. Images from abdominal computerized tomography (CT) scan revealed dilated, fluid filled appendix with surrounding inflammation and intra-operatively findings and histology confirmed the features of acute appendicitis (75).

A case of phlegmonous ileocolitis has been described in a 6 years old boy presented with acute onset of high-grade fever, vomiting, abdominal pain and diarrhea and history of exposure to COVID-19. Infectious screening confirmed positivity for COVID-19 immunoglobulin G (IgG) with negative nasopharyngeal PCR. The abdomen and pelvis CT scan showed phlegmonous changes around the ileocecal junction and terminal ileal loops with thickening and abnormal enhancement of terminal ileum loops, caecum and ascending colon, multiple mesenteric enlarged lymphnodes in the right lower quadrant and thickening of appendix. Repeat CT scan after 7 days of treatment with ceftriaxone, metronidazole, methylprednisolone and immunoglobulin, showed complete disappearance of the ileocecal phlegmon (76).

Intussusception has also been reported to be part of the clinical spectrum of COVID-19 in childhood. Several cases of infants with intussusception have been found to be positive for SARS-CoV-2. All cases had few days history of vomiting, jelly stool and abdominal pain prior of intussusception, however only 1 out of 5 infants had history of non GI symptoms such as coryzal symptoms and bilateral conjunctivitis and none of them presented fever, cough or dyspnea. One infant developed a progressive deterioration with respiratory distress and abdominal distension 2 days after pneumatic reduction of intussusception. Chest and abdomen CT scan showed infiltrates in both lung and peritonitis, ascites and swelling of small bowel wall. It has been postulated that SARS-CoV-2 might be an underlying cause of intussusception, as previously suggested for other viruses such as adenovirus and rotavirus (77). SARS-CoV-2 may play a role as well. Therefore, it seems paramount to exclude COVID-19 as a potential underlying cause of intussusception during this pandemic (78–81).

Pneumatosis intestinalis and protein loosing enteropahy has also been reported as acute manifestation of COVID-19 in a 6 years old boy tested positive for nasopharyngeal SARS-CoV-2 PCR. Abdomen CT scan performed for severe abdominal pain and right lower quadrant tenderness revealed pneumatosis in the ascending colon with dilated colon caliber (82).

A diffuse mesenteric lymphadenopathy has been reported as presentation of COVID-19 in an adolescent. Although no tissue sample proved that the mesenteric adenopathy was secondary to COVID-19, the child tested positive for SARS-CoV-2 by PCR from sputum, developed chest CT findings and hyperinflammatory response consistent with COVID-19 and other causes of mesenteric lymphadenopathy were ruled out (83).

GI symptoms have characteristically associated with the presentation of SARS-CoV-2-related multisystem inflammatory syndrome in children (MIS-C, described elsewhere), a condition characterized by systemic hyperinflammation with fever and multisystem organ dysfunction, which can potentially be confused with other common GI infections and even with IBD (59, 84, 85).

The prognosis of children with COVID-19 is generally excellent compared to older population or those with significant comorbidities. Children generally have milder symptoms and less likely need hospitalization (86). The need for intensive care support is low (3.3%) (87). Among 371 pediatric cases only 5 developed severe and critical illness and required intensive care (48).

## Impact of COVID-19 on Pediatric Gastroenterology

The COVID-19 pandemic and the subsequent infection control measures imposed by the governments have had a deep impact on healthcare delivery, not just on those affected by SARS-CoV-2 infection. The interruption of elective or routine clinical care, resetting of clinical priorities, lack of evidence regarding risk of COVID-19 and precautions taken during the pandemic in patients with chronic medical conditions have raised anxiety among patients and clinicians with a subsequent increase in burden on health-care systems and well-being of all patients.

The repercussions on pediatric gastroenterology practice have been significant. Physicians have faced unique challenges during COVID-19 pandemic, including the prevalence of GI symptoms, the possibility of a fecal-oral transmission, the uncertain risks of procedures, such as endoscopy, and the increased psychosocial burden reported during the pandemic on functional disorders.

Children with preexisting digestive diseases need appropriate and timely care during the pandemic. The challenge has been to continue providing essential care for these patients, while keeping safe both health care professionals and patients. In this regard, strategies such as an expansion of telemedicine tools, formal risk management of patients and the clinical pathways and appropriate prioritization of interventions have been implemented, in order to provide care for children with underlying gastrointestinal symptoms.

Below we outline the main challenges related to the practice of pediatric gastroenterology during COVID-19 pandemic.

### Impact of COVID-19 on Pediatric Chronic Gastrointestinal Diseases

There have been concerns expressed early in the COVID-19 pandemic, about the repercussions of children with chronic digestive disorders, such as those with IBD. These children tend to have more extensive and severe phenotype than in adults, with consistently higher need for immunomodulators and biologics. This vulnerable group requires regular follow-up, adequate nutrition, psychosocial support, and endoscopic and histological reassessment for paving an adequate management. Furthermore, the use of IBD-related immunosoppressive treatment has raised concerns regarding the possible higher risk of COVID-19 in these children with potential implications for their management and treatment.

The first provisional report from adults with IBD was reassuring (88). As the pandemic has progressed, the impact of COVID-19 on large cohorts of IBD patients has been brought to light through studies demonstrating the challenges in managing the IBD patients during this period.

The current observational evidence supports that children with IBD do not carry a higher risk of SARS-CoV-2 infection compared to the general population. Few cases of COVID-19 in children with IBD have initially been reported all showing a mild disease course, despite being treated with immunosuppressive medications (89). The evidence of association between increased levels of cytokines and chemokines, such as interleukin-6, with increased COVID-19 disease severity, in both adults and children, suggests that immunomodulatory therapive and passive immunization strategies could potentially protect from severe COVID-19 disease (90).

An observational study of a total of 522 IBD patients including 59 (11%) children was conducted in an Italian tertiary referral center between February and March 2020. The study reported no case or admission for SARS-CoV-2 infection in the IBD group compared to 479 patients without history of IBD admitted to the hospital for COVID-19 over the same period (91). The same author demonstrated a SARS-CoV-2 seroprevalance of 21% among 90 IBD patients, both adult and children, on biologic treatment. Interestingly no pediatric case tested positive and a 5-fold increased risk of positive result was found in older compared to younger age. The comparison with an aged-stratified control group failed to show any significant difference in terms of seroprevalence. Moreover, the COVID-19-related symptoms reported were all mild in this group (92).

Surveillance Epidemiology of Coronavirus Under Research Exclusion for IBD (SECURE-IBD) has assessed the impact of COVID-19 on both adult and children with IBD. In the first instance a total of 525 patients (median 41, range 5 to >90) were included in the SECURE-IBD database. Among the 29 patients <20 years old only 3 (10%) cases required hospitalization with no patient requiring intensive care. Reassuringly, no death occurred in the pediatric subgroup.

Risk factors associated with more severe COVID-19 and adverse outcome were older age, number of comorbidities, use of corticosteroids and 5-aminosacylitate (5-ASA)/sulfasalazine.

Surprisingly, biologic treatment with tumor necrosis factor (TNF) antagonist was not an independent risk factor for COVID-19. Actually TNF antagonist use was inversely associated with the endpoint of hospitalization or death, instead use of corticosteroids and age were positively associated with the outcome of death. The results drawn attention to the importance of steroid-sparing treatments for maintenance remission in IBD patients and the reassurance of use of TNF antagonist through COVID-19 pandemic (93).

The SECURE-IBD registry has also reported a rate of 7% hospitalization, 2% ventilation support requirement and no death in a pool of 209 COVID-19 cases in pediatric IBD. Risk factors for hospitalization included other comorbidities, use of sulfasalazine/mesalamine and steroid, moderate or severe IBD disease activity, and presence of gastrointestinal symptoms.

This database also confirmed the decreased risk for hospitalization associated with TNF antagonist monotherapy and the increased risk associated with sulfasalazine/mesalamine accordingly to the results reported in adults IBD (94).

Subsequently the impact of both monotherapy and combination of different medication classes on the risk of COVID-19 has been investigated in 1,439 adults patients with IBD. Thiopurine monotherapy and combination therapy with TNF antagonists were both significantly associated with severe SARS-CoV-2 infection requiring ICU admission, mechanical ventilation and/or death, compared with TNF antagonist monotherapy. The risk associated with combination therapy seems to be driven by thiopurines. No significant difference in COVID-19 outcome was found when comparing classes of biological treatments including TNF antagonist, IL-12/23 and integrin antagonists (95).

In contrast to the likely small primary clinical impact of COVID-19, there is a significant secondary impact on children with IBD. Ashton et al. (96) reported that the clinical pathways for the diagnosis of pediatric IBD have been hugely distorted by COVID-19 pandemic. Authors commented that more than 50% of suspected diagnosis of IBD during this period was diagnosed without a histological assessment due to restriction placed on endoscopy services in the UK centers. However, despite the aforementioned challenges, the care for known IBD patients, including biologic treatment infusions, consultations and surgery, if needed, had been largely maintained (96).

Martinelli et al. (97) reported a 64.2% reduction of hospital admission including biological infusion, new diagnosis, outpatient and inpatient appointments in a cohort of 180 IBD children during the first lockdown. Telephonic interviews revealed presence of symptoms suggesting a disease flare-up in up to 6.9%, suspension of immunomodulator treatment in 7% and delay of biological infusions in 18.1% (97).

In this respect it is noteworthy to highlight the possible consequences of the reduction of access to healthcare service during COVID-19 pandemic such as increase risk for delayed diagnosis and severe illness presentation even of usually benign gastrointestinal disorders. It is emblematic the two cases of coeliac disease (CD) presented with coeliac crisis, a life-threatening condition which is almost completely disappeared in developed countries, due to reduce access to healthcare service or fear to attend hospital (98, 99).

Conversely the restriction measures and lockdown has had a silver lining on adherence to gluten-free diet in children with CD. It has been showed that the compliance to gluten-free diet was unchanged in 70% and stricter in 29% of CD children during the lockdown. As hypothesized the positive outcome of dietary treatment was the result of not eating away from home, more time to prepare food, care for the child and control the adherence (100).

As different viral and coeliac biological factors could potentially interact, it has been hypothesized that CD patients may have a higher degree of susceptibility to SARS-CoV-2 infection or predisposition to more severe infection. To date only one study has explored the prevalence of COVID-19 in pediatric population with coeliac disease. Out of 387 children with CD recruited, 23 (5.9%) children reported COVID-19 like symptoms but none of them had a laboratory diagnosis of COVID-19 using a nasopharyngeal swab for real time RT-PCR. There were no complications reported in the supgroup of patients who experienced COVID-19 like symptoms and there was no significant increase in the prevalence of COVID-19 compared to the general population (101, 102).

Children with chronic GI disorders may face specific challenges brought by the pandemic and the enforced infection control measures. The restrictive measures to control the spreading of the infection may have driven major changes in social behaviors and affected the social health.

This is worthy of further attention, if we consider children with FGIDs, in whom the psychosocial factors play a crucial role in their development (103).

Although there are no data on the subject, it seems intuitive that children with FGIDs may be affected by pandemic lockdown measures in multiple ways. The disruption of routine, lack of social contacts, unfamiliar distance learning and worries about the school exams, may have worsened their symptoms. Moreover, the home environment in some families may have deteriorated with increased stress driven by factors such as financial insecurity, uncertainity about future health and increase in domestic abuse, which are known predisposing factors. On the other hand, in some children these measures may have had paradoxical positive effects, which may be related to the reducing stressful events, such as school, as well as to an increased parental closeness. Satisfying social functioning may have favorably influenced the occurrence of GI symptoms and the related FGIDs. For example, children who refuse to use the school toilets, a contributing factor in development and perpetuation of constipation, may have benefited from the increased time spent at home and the supervision from their family.

As the COVID-19 continues to spread around the world enforcing a dramatic long-term lifestyle change to conform to the infection control measures, there is a potential for secondary harm through inadequate diagnoses, undertreatment and delay in care delivery. This warrants the need for improvement of care in several areas such as virtual telehealth appointments, triage of patients using a multidisciplinary approach, appropriate use of COVID-19 secure or low risk facilities, and utilization of clinical networks to maintain high standard and quality of care for children with chronic gastrointestinal diseases.

### Challenges of Pediatric Gastroenterologists in the COVID-19 Era

Delivery of care in pediatric gastroenterology has considerably changed in the era of COVID-19, and one of the most affected fields has been the diagnostic GI endoscopy. Pediatric endoscopy procedures are considered at high risk of inhalation of airborne droplets and mucosal contact, hence carrying a high risk for COVID-19 transmission. All endoscopic procedures involve risk of generating aerosol and microdroplets by the instruments, valves, ports and air pressures during insufflation and suction, and also with the insertion and removal of instruments through the biopsy channel (104, 105).

Upper GI endoscopy is thought to carry a higher risk of aerosols than lower GI endoscopy due to prolonged contact with oropharyngeal secretions, including potential coughing and retching. Moreover, the majority of pediatric endoscopic procedures are performed while using general anesthesia, which increases the risk of droplet formation itself (106). Lower endoscopy involves exposure to feces, therefore possible fecal-oral transmission from extensive splashing of body fluids, including feces, during procedure (107). Both types of procedures involve short physical distance between patients and personnel, further increasing the risks of transmission from whichever route. Finally, with the increased prevalence of minimally symptomatic or asymptomatic children with COVID-19, risk stratification without pre-procedure nasal-pharyngeal swabbing is challenging, especially as the potential of SARS-CoV-2 transmission before symptoms manifest as well as the peri-endoscopic transmission of COVID-19 is well-documented (58, 108–111).

All these factors have led to restriction in pediatric GI services, especially for non-essential endoscopic procedures, in order to minimize avoidable transmission and undertake measures to assess the level of risk of virus transmission (112). One way to stratify the risk is based on known negative COVID-19 test results prior to the procedure. However, caution is necessary in applying this strategy as a false negative rate over 30% has been repeatedly described (113–117). Pre-screening of the pediatric patients using symptoms questionnaires is not useful, as children can present with either no symptoms or mild symptoms in up to 55% of cases (58, 62, 65, 86, 108, 118, 119). Therefore, all patients undergoing pediatric endoscopy may need to be considered at “high risk.”

The North American Society for Pediatric Gastroenterology Hepatology and Nutrition (NASPGHAN) has formulated a detailed position statement for the delivery of pediatric endoscopy in the era of COVID-19. Recommendations on how to provide services and minimize the risk to patients, caregivers and healthcare staff, while preserving supplies of critical personal protective equipment (PPE), have been formulated. Appropriate use of PPE is essential to minimize transmission of COVID-19 during pediatric endoscopic procedures and to preserve supply. It has also been provided recommendations on how to prioritize pediatric endoscopic procedures (120) (Table 3).

**Table 3:** Risk stratification of pediatric endoscopic procedures during COVID-19 pandemic [adapted from NASPGHAN position paper by Walsh et al. (120)].

Emergent
Endoscopic procedure for intervention and/or diagnosis of potentially life-threatening conditions and/or for conditions where if left untreated has significant morbidity/mortality. Emergent procedures should continue.
Urgent
Endoscopic procedure for which findings can change management significantly and/or intervention for stable patient which is not immediately life-threatening, but can lead to significant complications if delayed. Urgent procedure should be paused. Risk and benefit of this decision should be weighted.
Elective
Endoscopic procedure that can be post-poned and/or managed alternatively; encompasses conditions not considered emergent or urgent. Elective procedure should be post-poned.

As there is a high risk associated with pediatric endoscopic procedures and a potential shortages of healthcare resources, PPE, staff through illness and self-quarantine, it is important to minimize endoscopic procedures to preserve PPE supply and limit exposure of endoscopic personnel. In spite of expert opinions and society recommendations, deciding whether or not to postpone an endoscopy is not always unambiguous, particularly in patients with known IBD or eosinophilic esophagitis, where endoscopic findings can guide patients' management. However, no clear indication is available for those endoscopies that cannot be considered urgent, but may be associated with unfavorable consequences if rescheduled in the long term, such as surveillance and efficacy assessment of therapies. The different epidemiology of pediatric GI disease, as compared with adult disease, may influence the need for emergent or urgent endoscopy. For instance, children present with greater overall disease severity of IBD, or need for enteral feeding devices for provision on nutrition support. Delay in diagnosis and/or management may have significant impact on patient's outcomes and growth, as well as leading to anxiety among patients, caregivers and staff. It is, therefore, important to closely follow children for whom procedures are delayed and ensure timely booking once the immediate impact of the COVID-19 pandemic has eased or passed.

While recommendations for endoscopic procedures in children have been provided, no recommendations have been provided for GI motility tests such as manometry (esophageal, antroduodenal, colonic and anorectal), pH-impedance and breath testing, which have automatically been either stopped or limited acknowledging their non-urgent status. Conversely, recommendations for motility tests during COVID-19 pandemic have only been provided in adults (121).

As with other medical disciplines, the outbreak of the COVID-19 pandemic has also had a dramatic impact on outpatient pediatric gastroenterology provision. Routine face-to-face contact poses an increased risk of exposure and potential viral transmission, even during brief ambulatory interaction. It has been found that SARS-CoV-2 causes significant environmental contamination in the hospital rooms of COVID-19-confirmed patients (122). It has been shown that SARS-CoV-2 can be detected in two-third of the personal air samplers worn by staff who maintained a distance of > 6 feet from patients (123).

Accordingly the use of remote telehealth/videohealth delivery of care, where appropriate, is now a crucial tool for the pediatric gastroenterologists. Through telehealth, patients can stay home, minimizing exposure to higher risk environments such as hospitals or clinics, and still have a virtual consultation with their physicians, while the latter can encompass remote clinical assessment and treatment optimisation and provide safely care without increasing risk for both the healthcare staff, patients, and their family. Additionally, it allows for identification of those children requiring a face-to-face assessment or diagnostics.

## COVID-19 Pandemic Experience in a Quaternary Pediatric Gastroenterology Service

The impact of COVID-19 on our patients and on the provision of pediatric gastroenterology services during the pandemic is likely to mirror that of other units internationally.

The pediatric gastroenterology unit of Great Ormond Street Hospital (GOSH) provides specialized GI services to children in North and Central London, as well as quaternary commissioned services for conditions such a pediatric intestinal pseudo-obstruction, to the rest of the United Kingdom. Based on the impact of COVID-19 on our colleagues, in both adult and pediatric care, hospital services within London urgently reconfigured its services to ensure adequate access to intensive care facilities and adequate inpatient beds for those with moderate or severe COVID-19. Consequently, our unit, became the main pediatric center for all children within North and Central London from March 2020.

To provide safe access to specialist GI investigations, such as endoscopy, and management of acute flares of chronic digestive disorders, such as IBD, the unit worked at pace to quickly establish new clinical networks with surrounding hospitals, including other pediatric gastroenterology units, where staff were redeployed to support adult services. Through collaboration, staff re-deployment and implementation of altered working practices, the equity of access to specialized pediatric care was safeguarded.

**Figure 3:**
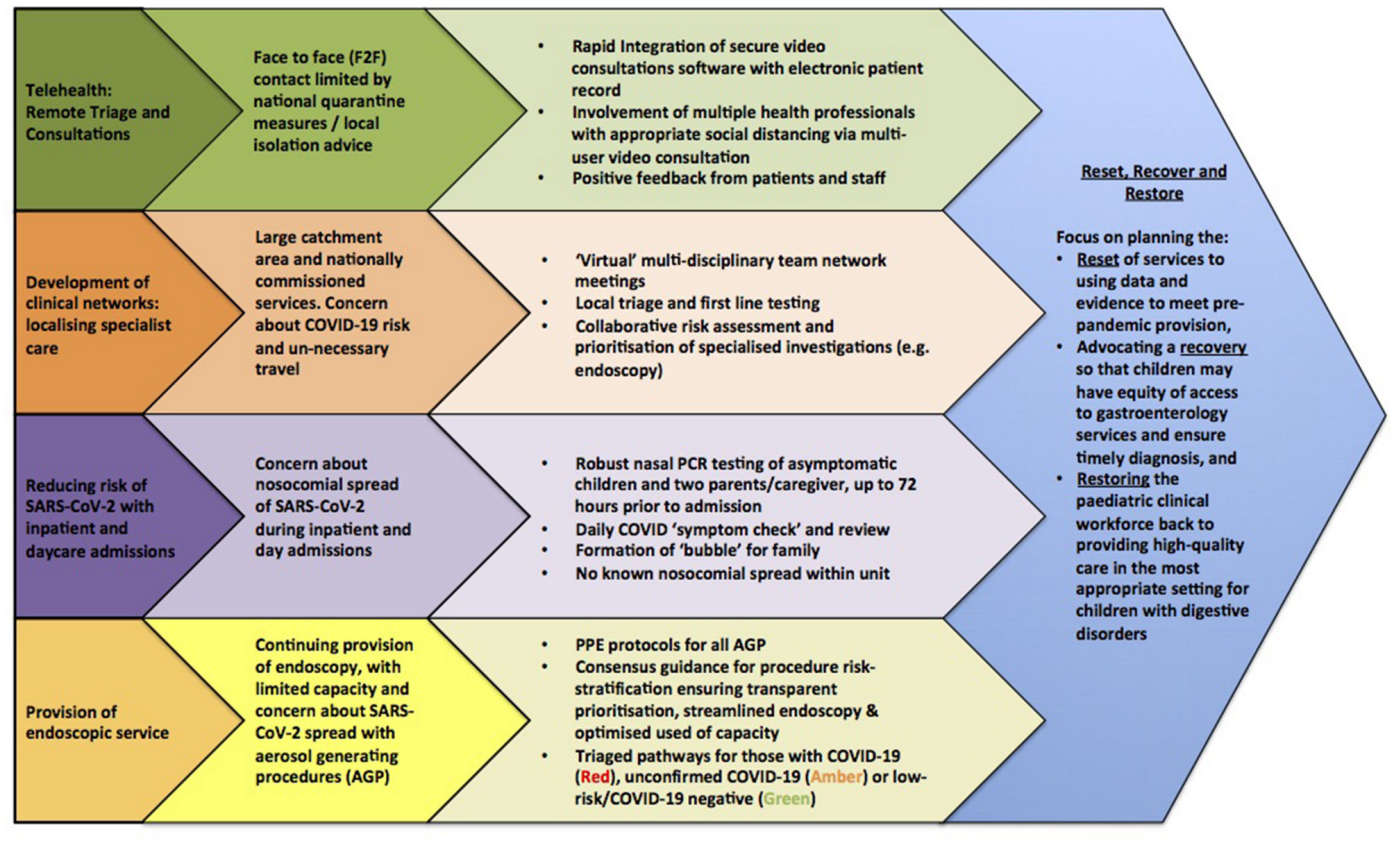
Strategies for managing gastrointestinal conditions during COVID-19 pandemic.

We have outlined the strategies implemented during COVID-19 pandemic in Figure 3.

National infection and prevention control recommendations have been used to guide returning services (124, 125)^1^.

## Conclusion: Emerging Needs and Future Implications

COVID-19 enteric infection and its impact on the GI tract is cleary demonstrated, yet whether the intestinal epithelial cells are primarly infected with SARS-CoV-2 *via* the oral-fecal route or whether it is secondary to respiratory infection still remains unclear.

The current data suggest a high prevalence of GI symptoms in children raising the need to include SARS-CoV-2 infection among the differential diagnosis of acute diarrhea and/or vomiting, at least for the duration of the pandemic. The atypical GI manifestations of SARS-CoV-2 infection could be a diagnostic challenge. Given the ongoing emergency of GI manifestations of the disease, a high index of suspicion should be kept for COVID-19 in all children presented with GI symptoms.

At present, although the major burden of COVID-19 is represented by the disease manifestation of infected children, the impact on the management and diagnosis of children with chronic GI diseases is also significant. Pediatric gastroenterologists and allied health care professionals continue to learn and adapt to this novel viral infection, which affects all aspects of healthcare. Although, there is still no clear evidence about a fecal-oral transmission, the potential risk of this route impacts the delivery of healthcare such as endoscopy.

Additionally, major challenges face patients and physicians brought by restrictive measures adopted by governments to control the pandemic that have considerably changed healthcare systems. However, professionals have scarce high-grade evidence to base clinical and operational decisions for children with chronic digestive disorders during COVID-19 pandemic. Available evidence suggests children with chronic GI disease do not have higher risk of severe SARS-CoV-2 infection, however the evidence is largely observational and further studies assessing the outcome of COVID-19 in this group of children are required. Furthermore, monitoring the impact of the COVID-19 pandemic on diagnosis, management and care delivery in pediatric chronic GI disorders is required to ensure that the access to care and preventing harm to patients is quantified, prior to restoration of normal service provision. In the meantime, it is essential to implement remote telehealth services, guidelines on diagnosis and management during COVID-19 and measures that minimize the risk of transmission in healthcare settings.

## Author Contributions

OB: guarantor of manuscript and conception of the manuscript, critical revision of the manuscript, and approval of the final version of the paper. MP: preparation of synopsis data, drafting the article, and approval of the final version of the paper. AR: preparation of synopsis data, drafting the article, critical revision of the manuscript, and approval of the final version of the paper. FK: preparation of synopsis data, critical revision of the manuscript, and approval of the final version of the paper. EG: conception of the manuscript, critical revision of the manuscript, and approval of the final version of the paper. All authors contributed to the article and approved the submitted version.

## Conflict of Interest

The authors declare that the research was conducted in the absence of any commercial or financial relationships that could be construed as a potential conflict of interest.

## Footnotes

^1^Available online at: www.healthcareitnews.com/news/emea/uks-great-ormond-street-hospital-children-nhs-ft-becomes-first-hospital-europe- attain.

## Abbreviations

SARS-CoV-2: acute respiratory syndrome coronavirus 2
COVID-19: coronavirus disease 2019
GI: gastrointestinal
SARS: acute respiratory syndrome
ACE-2: angiotensin-converting enzyme-2
TMPRSS2: type II transmembrane serine protease
RT-PCR: reverse-transcriptase polymerase chain reaction
INF: interferon
IL: interleukin
IBD: Inflammatory bowel disease
CNS: central nervous system
ENS: enteric nervous system
FGIDs: functional gastrointestinal disorders
CT: computerized tomography
IgG: immunoglobulin G
MIS-C: multisystem inflammatory syndrome in children
SECURE-IBD: Surveillance Epidemiology of Coronavirus Under Research Exclusion for inflammatory bowel disease
5-ASA: 5-aminosacylitate (ASA)
TNF: tumor necrosis factor
CD: coeliac disease
PPE: personal protective equipment
GOSH: Great Ormond Street Hospital.
